# The role of microglia in viral encephalitis: a review

**DOI:** 10.1186/s12974-019-1443-2

**Published:** 2019-04-09

**Authors:** Zhuangzhuang Chen, Di Zhong, Guozhong Li

**Affiliations:** Department of Neurology, The First Affiliated Hospital, Harbin Medical University, 23 You Zheng Street, Harbin, 150001 Heilong Jiang Province People’s Republic of China

**Keywords:** Microglia, Viral encephalitis, IFN-1, P2Y12, Aβ, Autophagy

## Abstract

Viral encephalitis is still very prominent around the world, and traditional antiviral therapies still have shortcomings. Some patients cannot get effective relief or suffer from serious sequelae. At present, people are studying the role of the innate immune system in viral encephalitis. Microglia, as resident cells of the central nervous system (CNS), can respond quickly to various CNS injuries including trauma, ischemia, and infection and maintain the homeostasis of CNS, but this response is not always good; sometimes, it will exacerbate damage. Studies have shown that microglia also act as a double-edged sword during viral encephalitis. On the one hand, microglia can sense ATP signals through the purinergic receptor P2Y12 and are recruited around infected neurons to exert phagocytic activity. Microglia can exert a direct antiviral effect by producing type 1 interferon (IFN-1) to induce IFN-stimulated gene (ISG) expression of themselves or indirect antiviral effects by IFN-1 acting on other cells to activate corresponding signaling pathways. In addition, microglia can also exert an antiviral effect by inducing autophagy or secreting cytokines. On the other hand, microglia mediate presynaptic membrane damage in the hippocampus through complement, resulting in long-term memory impairment and cognitive dysfunction in patients with encephalitis. Microglia mediate fetal congenital malformations caused by Zika virus (ZIKV) infection. The gene expression profile of microglia in HIV encephalitis changes, and they tend to be a pro-inflammatory type. Microglia inhibited neuronal autophagy and aggravated the damage of CNS in HIV encephalitis; E3 ubiquitin ligase Pellino (pelia) expressed by microglia promotes the replication of virus in neurons. The interaction between amyloid-β peptide (Aβ) produced by neurons and activated microglia during viral infection is uncertain. Although neurons can mediate antiviral effects by activating receptor-interacting protein kinases 3 (RIPK3) in a death-independent pathway, the RIPK3 pathway of microglia is unknown. Different brain regions have different susceptibility to viruses, and the gene expression of microglia in different brain regions is specific. The relationship between the two needs to be further confirmed. How to properly regulate the function of microglia and make it exert more anti-inflammatory effects is our next research direction.

## Background

Encephalitis is a common and serious disease, and its clinical manifestations vary from person to person. Its main characteristics include altered mental status and various combinations of acute fever, seizures, neurologic deficits, cerebrospinal fluid (CSF) pleocytosis, and abnormalities in electroencephalographic (EEG) [1]. Although there are many reasons accounting for this disease, the most commonly identified causes are neurotropic viruses. Every year in the USA, there are about seven patients per 100,000 population diagnosed as encephalitis. Among all the cases, approximately half of them have unknown reasons. Of the cases with a known cause, viruses represent 20–50%. Herpes simplex virus (HSV) takes up 50 to 75% of identified viral cases, with varicella–zoster virus (VZV), enteroviruses, and arboviruses accounting for the majority of the remainders [2]. It is estimated that the median hospitalization charge for a patient with viral encephalitis is $89,600 for West Nile virus encephalitis and $58,000 for HSV encephalitis. Each year in the USA, approximately 6000 patients are hospitalized for acute viral encephalitis. The total annual cost is up to $350 million to $540 million, without counting the cost of care after discharge, costs for family caregivers, and lost earning [3].

Microglia are CNS-resident mononuclear phagocytic cells characterized by a unique ramified shape and distinctive gene expression [4]. Most microglia are derived from a yolk sac progenitor and seed the brain early in development [5]. As resident cells, microglia are assumed to help orchestrate the immune response to pathogen infection of the brain. While often touted as immune sentinels, little is known about how or if microglia engage in this function [6]. So far, a large body of evidence stemming from both in vitro and in vivo studies have indicated that microglia are capable of responding to viral PAMPs [7, 8], but the multifaceted function of microglia involved in viral encephalitis needs further understanding. Recently, how microglia are recruited around infected neurons and the interaction between microglia and other cells has become a hot topic. In this review, we will talk about the function of microglia in viral encephalitis.

## Microglia play an anti-inflammatory role in viral encephalitis

### Microglia are recruited around the infected neurons by sensing extracellular ATP signals

**Fig. 1:**
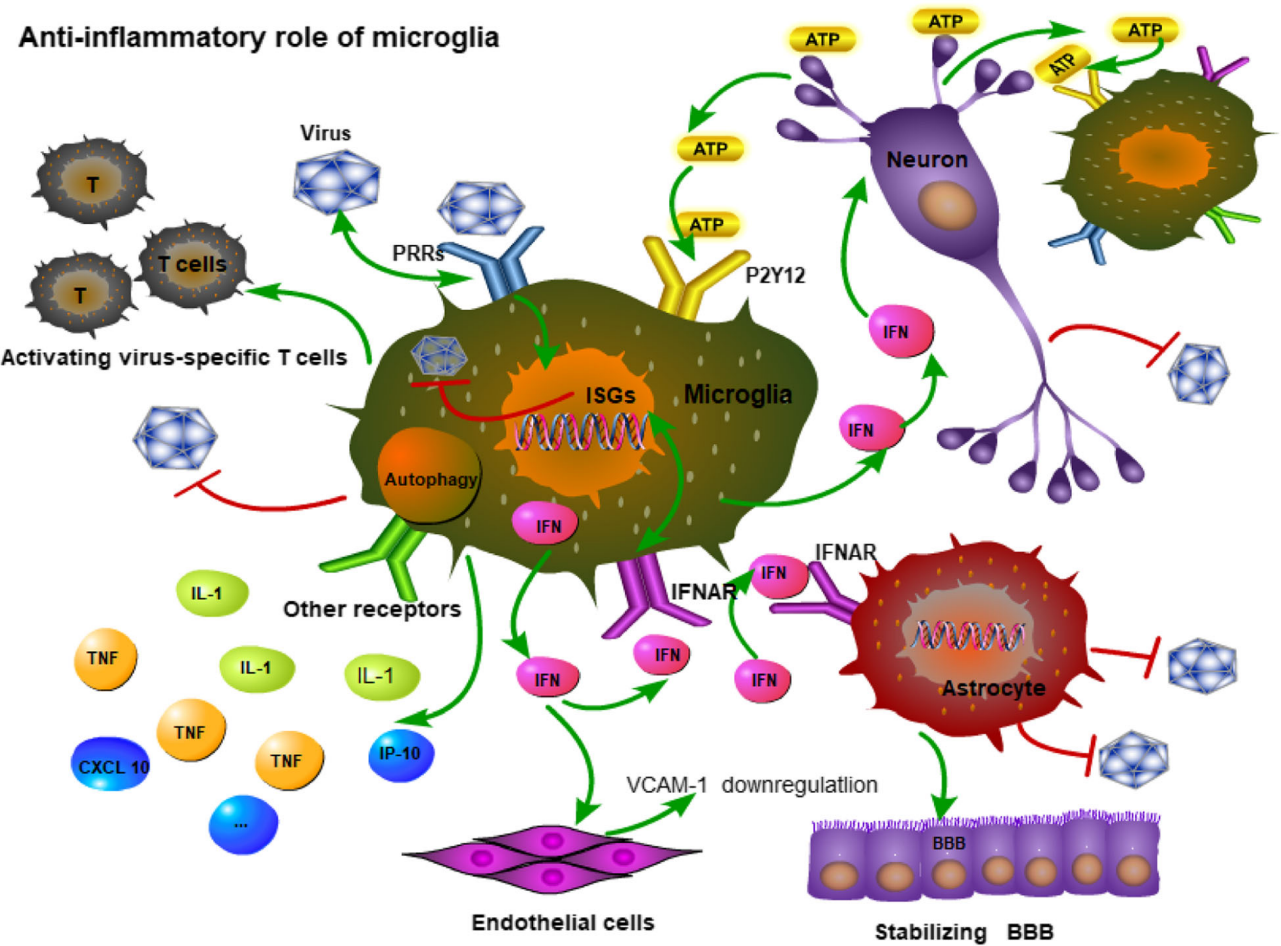
Illustration of the anti-inflammatory role of microglia in viral encephalitis. The picture shows that microglia can sense ATP signals through the purinergic receptor P2Y12 and are recruited around infected neurons to exert phagocytic activity. Microglia can exert a direct antiviral effect by producing type 1 interferon (IFN-1) to induce IFN-stimulated gene (ISG) expression of themselves. IFN produced by microglia exects indirect antiviral effects by acting on other cells to activate corresponding signaling pathways

Microglia can sense the nucleotides released by infected neurons and are rapidly recruited around infected neurons to exert phagocytic activities in the brain (see Fig. 1). Neurons release more ATP after viral infection, while the levels of ATP, ADP, AMP, and adenosine in cell lysates decrease. Noxious stimuli in neurons trigger a sustained increase in extracellular ATP, which causes microglia to become activated and recruited in minutes to hours [9]. ATP is a potent agonist of the P2Y G protein-coupled receptor [10]. Studies have shown that P2X7 or P2Y12 are both abundant in microglia, and P2Y12 is a microglia-specific marker in the brain [11].

Extracellular ATP is a strong chemotactic signal for microglia. By stimulating the P2Y receptor of microglia, it mediates the rapid response of microglia to the injury site [12]. The number of P2Y12 receptors on the surface of microglia increased over twofold in response to viral infections. Some experiments have built a P2X7- or P2Y12-deficient mice model (P2X7^−/−^ or P2Y12^−/−^) in vivo, the lack of P2X7 has no effect on the recruitment of microglia, whereas an absence of P2Y12 resulted in> 50% reduction in the numbers of microglia recruited to infected neurons. This indicates that microglia are recruited around the infected neurons via P2Y12 signaling. The CD86+ phagolysosomes of P2Y12^−/−^ microglia decreased significantly compared with normal mice which indicates that P2Y12 is essential for microglia to exert phagocytic activity [9]. Microglia are key contributors to the recruitment of monocytes into the brain during viral infection. In microglia-depleted mice, the phenomenon of monocytes entering the brain is almost completely gone. CD45+ blood-borne leukocytes were seen in the brain of P2Y12^−/−^ mice after infection, and mononuclear cell infiltration was not impaired, which indicates that microglia recruit monocytes through a P2Y12 independent mechanism [9].

Analysis of temporal lobe specimens in patients with herpes simplex type 1 (HSV-1) encephalitis shows that P2Y12-positive microglia processes extend to HSV-1 positive cells, and there are about 1–3 activated microglia around each infected neuron. Microglia appear around the cell bodies and dendrites of neurons before the mature virus particles appear in infected neuronal cells. At this stage, the neuronal membrane is intact and there is a normal chromatin structure in the nucleus. Cell trajectory analysis has shown that the speed of microglia movement in mice with viral encephalitis is reduced, which is related to their tendency to stay around the infected cells [9]. Typical neurological symptoms appear in mice infected with virus when microglia are completely absent, but not P2Y12 deficiency [9]. Consistent with this, mice with microglia depletion have higher viral loads and succumb to infection. The rapidly deteriorating neurological symptoms in microglia-depleted mice may be associated with significantly increased neuronal infection and potential microglial protective mediators such as interleukin-10 deficiency [13].

### Microglia play a direct antiviral effect by producing IFN-1 after recognition of virus by pattern recognition receptors (PRRs)

Mouse hepatitis virus (MHV) is a neurotropic coronavirus. On the fourth day of viral infection, microarray analysis of infected mouse microglia showed that the IFN-1 pathway was the most upregulated [14]. IFN-1 protects the host because treatment with exogenous IFN-α or IFN-β limits viral replication, whereas the infection of mice with IFN-1 signaling deficiency can convert nonfatal coronavirus to a lethal type [15]. In the mouse model of vesicular stomatitis virus (VSV) encephalitis, it was found that infected microglia produced IFN-1, and both infected and uninfected microglia upregulated the expression of IFN regulatory factors 7 (IRF7) and activated innate immunity which limits the transsynaptic spread of VSV [16]. Viral infection can induce the expression of IFN-stimulated genes (ISGs), thereby interfering with viral replication and promoting viral clearance [16] (see Fig. 1).

Microglia express a wide range of PRRs, which are important for the defense of HSV-1. Virus induces expression of IFN-1 by the recognition of nucleotides and (RNA-/DNA-sensing) PRRs [7, 8] (see Table 1 for a summary). The combination of IFN-1 and the heterodimeric receptor IFNAR produces a cellular response that initiates a signaling cascade that promotes heterodimers STAT 1/2 nuclear translocation and transcriptional activation of ISGs [17]. The rapid expression of hundreds of ISGs is critical for controlling viral infections because these proteins block the entry, translation, transcription, assembly, and efflux of the virus [18]. Cytosolic dsDNAs can induce a strong innate immune response. GMP–AMP synthase (cGAS) is a cytosolic DNA receptor [19]. The binding of cGAS to cytosolic DNA produces a second messenger, cyclic-GMP–AMP (cGAMP), which activates downstream STING and ultimately activates transcription factor IRF3, which promotes IFN-β production [8, 20].

**Table 1:** Summary of receptors of microglia involved in viral encephalitis

Articles	Recognition	Receptors’ classification	Location	Effects
Paludan et al. [19]	DNA sensors	TLR3/9; cGAS	Endosomes; cytoplasm	Leading to activation of IRFs and induction of IFNsStimulating autophagy and different types of cell death Activating the inflammatory NF-kB and inflammasome pathways
Mogensen [21]	RNA sensors; surface glycoproteins (GP) sensors	TLR7/8RLRs (RIG-I, MDA5); TLR2/4	Endosome/cytoplasm; cell surface	Resulting in an antiviral response mediated by type I IFN and cytokine production
Sperlagh et al. [11]	ATP/adenosine sensors	P2X7/P2X4; A1/A2A/A3 adenosine receptors	Cell surface	Involved in secretion of various factors, such as IL–1β, IL–6, and TNF-α, mediating apoptosis Regulating the proliferation/survival of microglia
Fekete et al. [9]	ATP	P2Y12	Cell surface	Microglia are recruited around the infected neurons via P2Y12 signaling P2Y12 is essential for microglia to exert phagocytic activity.
Vasek et al. [52]	Complement C3 and C3 cleavage products	C3R	Cell surface	Damaging of presynaptic ends and resulting in visual-spatial processing disorders and impaired memory function

Different types of PRRs can be involved in the identification of viral infections, including membrane-associated TLRs, cytosolic RNA-sensing RIG-like receptors (RLRs), and DNA sensors. PRRS activates IFN regulatory factors 3 (IRF3) through unique adaptor molecules such as TIR domain-containing adaptor inducing IFN-β (TRIF), mitochondrial antiviral signaling (MAVS), and stimulator of type I IFN genes (STING) [21]. Mutations in the IRF3 gene in herpes simplex encephalitis (HSE) patients impaired interferon production and the TLR3-TRIF pathway is the most severely damaged [22]. The cGAS-STING and TLR3-TRIF pathways are the major intrinsic sensing pathways to control HSV1 infection in the CNS. Microglia can induce IFN responses by recognizing viral pathogen-associated molecular patterns (PAMPs) through PRRs. In a typical IFN-1 response pattern, PRRs first induce the production of IFN-β after recognition of PAMPs, and then IFN-β binds to IFN-α receptor (IFNαR) to initiate IRF7 gene expression in an autocrine or paracrine manner which enables a complete IFN-1 response in viral spread or secondary infections [23]. In order to simulate the natural pathway of HSV-1 entering the central nervous system by retrograde transport, the previous experiment used an ocular infection model. It was confirmed that microglia are the main source of HSV-induced IFN-1, which is induced in a cGAS-STING-dependent manner. Infected cells including neurons and microglia detected HSV-1 by the cytoplasmic DNA receptor, the adaptor protein STING. The brain of mice lacking cGAS and STING detected higher viral load, and the virus spread widely to the midbrain, hypothalamus, and preoptic area. However, studies have shown that the recruitment of microglia to the infection site is not related to STING [24].

In a model of MHV infection, depletion of microglia using colony-stimulating factor 1 receptor (CSF1R) inhibitors revealed that depletion of microglia at 0–6 days of infection would result in increased mortality and a later depletion had no influences on survival. It indicated that microglia play an important role in controlling viral replication and reducing mortality in the early stage of infection [14]. Upon intranasal vesicular stomatitis virus (VSV) infection, studies have found activated microglia and monocyte aggregation in the olfactory bulb. Studies have also shown that microglia accumulating around the olfactory bulb form a natural immune barrier plays an important role in limiting the spread of VSV in the CNS and preventing lethal encephalitis. The formation of the intrinsic microglia barrier is regulated by IFNAR signaling of neurons and astrocytes and is not regulated by microglia themselves [25]. It has been previously reported that the use of brain-penetrant CSF-1R inhibitor BLZ945 in mice can remove microglia [26]. IL-34, as a CSF-1R ligand, is essential for the maintenance of microglia. BLZ945 plays a role in specifically depleting microglia by inhibiting the interaction of CSF-1R with IL-34 [27]. After depletion of microglia, VSV-infected mice have higher viral load in the brain, cerebellum, and brainstem and have higher mortality, indicating that the formation of microglia barrier in the olfactory bulb is indispensable to limit the spread and spread of VSV [25]. Sall1 is specifically expressed in microglia and is a negative regulator of microglia activation. Sall1 is downregulated in microglia when infected by pathogens, thereby promoting microglia activation [28].

### IFN produced by microglia acts on other cells to exert indirect antiviral effect

Microglia confer STING-dependent antiviral activity to neurons and initiate the production of IFN-1 in astrocytes via the toll-like receptor 3 (TLR3) pathway [24]. Type I IFNAR signaling in astrocytes acts to stabilize the blood-brain barrier (BBB) and protect the brain from viral infections and immunopathology (see Fig. 1). In a mouse model of West Nile virus (WNV) infection, it was found that the permeability of the BBB increased in mice lacking IFNAR, especially in the hindbrain, which leads to an increase in viral entry, indicating that IFNAR signaling has important regulatory effects on BBB permeability in this brain region. The study found that human and mouse cerebellar astrocytes have higher expression of PRRs and ISGs in both steady state and IFN-induced state compared with cerebral cortical astrocytes. Cerebellar astrocytes can reach maximum ISG expression more quickly after receiving IFN stimulation [29]. In vitro studies have shown that IFNAR pathways in brain microvascular endothelial cells (BMECs) and astrocytes can both promote the integrity of tight junction. Studies have shown that IFN-1 stabilizes the integrity of BBB and promotes the formation of tight junctions by rearranging endothelial junction proteins via Rho-Rac signaling pathways, promoting BBB tightening and blocking the expression of IL-1β and TNF-α [30].

It is well known that vascular cell adhesion molecule 1 (VCAM-1) is a neuroinflammatory regulator on vascular endothelial cells [31]. IFN-1 regulates immune transport by regulating the expression of VCAM-1 (CD 106) on neurovasculature [32]. IFN-β treatment of either human or mouse primary astrocyte cultures from the cerebellum, but not from the cerebral cortex, significantly downregulates VCAM-1 expression [29]. Drug blocking of VCAM-1 binding to its ligand leukocyte adhesion molecule 4 (VLA-4) has been shown to reduce pathogenic neuroinflammation and improve survival after WNV infection [33]. In IFNAR-deficient mice, cerebellar infiltrating lymphocytes and macrophages were significantly reduced after treatment with VLA-4 blocker [29]. Loss of astrocyte IFNAR signaling pathway can cause a variety of consequences, including increased expression of inflammatory cytokines and chemokines, which disrupt the blood-brain barrier during neurotropic viral infection [30]. In different brain regions, transcription, immunophenotypic, and bioenergetic properties of microglia are heterogeneous. Cerebellar and hippocampal microglia maintain a stronger immune alert status than microglia in the striatum and cortex. The sensitivity of different brain regions to aging is different, and the cerebellum is the most obvious. This suggests that different brain regions have different responses to aging-related neurodegenerative lesions [34].

Microglia also affect the adaptive immune response. Depletion of microglia changes the response of CD4+ T cells to viral infection in the brain, the total number and percentage of CD4+ T cells decrease, and the frequency and number of Tregs also decrease, indicating that microglia are crucial for fully activating virus-specific T cells. Deletion of microglia in the early stages of WHV infection exacerbates infection, which may be associated with loss of microglial antigen presentation function, as microglia participates in antigen presentation by upregulating *Tap1* and *Itgax*. Depletion of microglia can also result in the loss of major MHC II-expressing cell type, which can also result in decreased expression of MHC II in incoming monocytes/macrophages. Because MHC II is required for re-stimulation and activation of CD4+ T cells, a decrease in MHC II reduces the response of virus-specific CD4+ T cells [14].

In addition to producing IFN-1, microglia can also produce other cytokines. The vitro cell culture demonstrates that cytomegalovirus (CMV)-infected astrocytes recruit microglia to the infected foci by releasing monocyte chemoattractant protein 1 (MCP-1). Microglia produces tumor necrosis factor α (TNFα) and interleukin 1β (IL-1β), interleukin 6 (IL- 6) exerting antiviral effect and inhibiting the replication of CMV in astrocytes [35]. CMV-infected microglia produce T lymphocyte chemotactic factor CXCL 10, recruiting activated T lymphocytes into the infected area, and T cells produce IFN-γ to inhibit virus replication and dissemination [36]. Even with limited viral replication, cytopathic effects were evident in the HSV-infected microglia. Microglia responded to nonpermissive viral infection by producing considerable amounts of TNF-α, IL-1β, and IP-10. TNF-α inhibits HSV replication in astrocytes, and IP-10 possesses direct antiviral activity in neurons [37].

### Microglia restrict viral infection by autophagy

Inflammation-induced, STING-dependent autophagy limits Zika virus (ZIKV) infection in Drosophila brains. The activation of NF-kB dependent on ZIKV induces expression of Drosophila stimulator of interferon genes (dSTING) in the brain. dSTING protects against ZIKV infection by inducing autophagy in the brain. Loss of autophagy leads to increased viral load in the brain and increases infection in Drosophila [38]. Multiple microbial infections activate NFkB-dependent inflammatory signaling cascades, which induce transcription of downstream antimicrobial effectors, including antimicrobial peptides (AMPs), thereby reducing or eliminating infection [39]. Endogenous, bacterial-derived circulating dinucleotides and increased expression of STING mRNA can activate STING [40]. From fruit flies to mammals, activation of antimicrobial autophagy protects cells against a variety of pathogens, including bacteria, viruses, and parasites [41, 42]. Studies have shown that Drosophila infected with ZIKV induces activation of NF-kB signaling in the IMD pathway, thereby activating downstream dSTING. dSTING can limit intracellular pathogens by inducing autophagy [43].

Autophagy protects neurons against a variety of stressors, including viral infections. Autophagy clears infection by capturing pathogens, including viruses, in the cell without causing cell death, which is beneficial for mature neurons [42, 44]. Previous studies used heat shock-inducible gal4 (HS-gal4) to silence autophagy core genes *Atg5* and *Atg7* in vivo [45] and found that the virus titer of ZIKV in Drosophila is significantly increased. Relish is a transcription factor of NF-kB in the IMD pathway. Using a validated RNAi line which specifically removes Relish from neurons or glial cells in vivo, known as a neuron-specific gal4 driver (elav-gal4) or a glia-specific gal4 driver (Repo-gal4), respectively [46], increased viral infection. In mammals, autophagy limits the replication or pathogenesis of herpes simplex virus type 1 (HSV-1) [47]. Intracellular receptors, such as retinoic acid-inducible gene-I (RIG-I) and melanoma differentiation-associated protein 5 (MDA-5), collectively referred to as RIG-I-like receptors (RLRs), can also detect cytoplasmic viruses nucleic acid [48]. Using the advantages of Drosophila, the study found that the PRR-autophagy axis plays an important role in host defense [49].

## Microglia cells play a pro-inflammatory role in viral encephalitis

### Microglia-mediated synaptic loss causes memory impairment

**Fig. 2:**
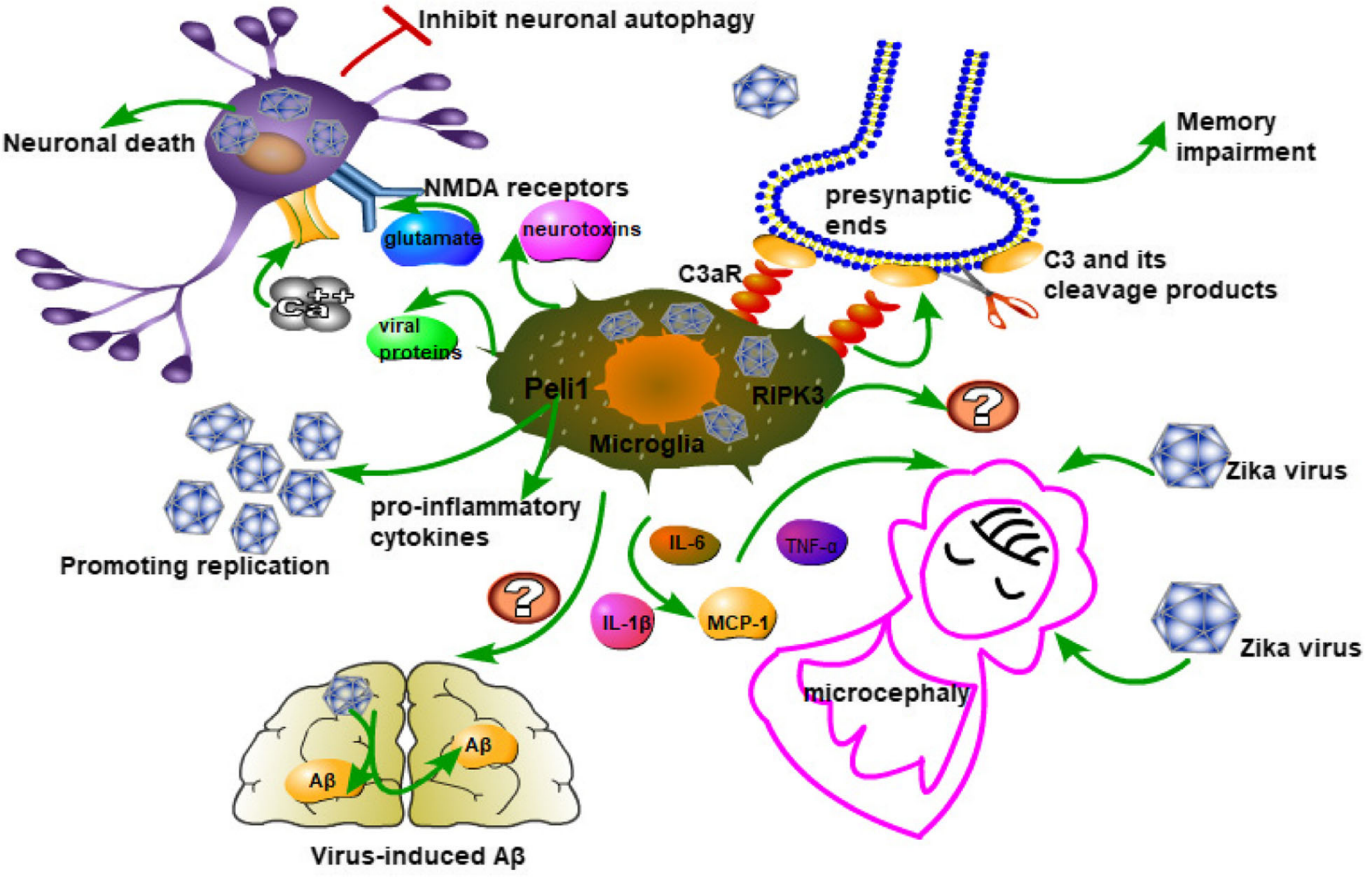
Illustration of the pro-inflammatory role of microglia in viral encephalitis. The picture shows some harmful effects of microglia in viral encephalitis, such as mediating presynaptic membrane damage, causing fetal congenital malformations, and exacerbating the damage of CNS in HIV encephalitis. Microglia play an uncertain role in virus-induced Aβ. The role of the RIPK3 signaling pathway in microglia in viral encephalitis remains obscure

Fifty percent of patients with neurotropic WNV infection show chronic cognitive sequelae [50]. Patients who survive after infection with WNV usually present with visual-spatial processing disorders and impaired memory function [50, 51]. Studies have shown that complement C3 and C3aR mediate the loss of presynaptic ends in mouse hippocampus (see Fig. 2). Mice demonstrate spatial learning in the recovery period of neurological invasive diseases in West Nile. Both neurons and microglia express C3aR, which recognizes C3 cleavage products. C3 and its cleavage products attract microglia to gather around the neurons to exert phagocytosis activity and to clear the presynaptic ends. In the situation of a neurotropic virus infection, clearance of the presynaptic end may prevent trans-synaptic spread of the virus and stop abnormal signals from infected neurons [52]. Infected and activated microglia in the CNS persist for more than 12 months after receiving antiviral therapy in patients with HSV encephalitis [53]. This may lead to chronic damage to the CNS. Microglia can produce TNFα [35, 36]. Local increase of TNFα in the hippocampal dentate gyrus activates astrocyte TNF receptor type 1 (TNFR1), which in turn triggers an astrocyte-neuron signaling cascade that results in persistent functional modification of hippocampal excitatory synapses which can lead to cognitive disturbance [54]. Glutamatergic gliotransmission provides a stimulatory input to excitatory synapses in the hippocampal dentate gyrus. TNFα critically controls the process which may influence synaptic transmission and plasticity [55]. This suggests that microglia may also affect synaptic function through cytokines.

### Microglia-mediated congenital malformation

The unprecedented re-emergence of the Zika virus (ZIKV) virus has shocked the world, and reports on microcephaly in Brazil are also increasing [56] (see Fig. 2). In Brazil, the incidence of fetal microcephaly in ZIKV-infected pregnant women is approximately 12.8% [57]. Surprisingly, there have been increased reports of cases of simultaneous diagnosis of Guillain-Barré syndrome in adults with French Polynesia ZIKV infection [58]. Recent evidence suggests a causal relationship between prenatal ZIKV infection and severe brain abnormalities including microcephaly [59].

ZIKV was detected in the brain of a microcephalic fetus [60]. This means that there is a strong connection between ZIKV and these neurological diseases. The study found that ZIKV mainly infects fetal microglia and induces high levels of pro-inflammatory mediators including IL-6, TNF-α, IL-1β, and MCP-1. These inflammatory factors may be harmful to the fetus [61]. Microglia’s perivascular localization enables them to survey the influx of blood-borne components into the CNS [62]. Thus, microglia are highly susceptible to ZIKV infection. The analysis of vitro culture and microcephalic fetal brain histology showed that ZIKV leads to activation of microglia [60]. This, in turn, causes localized neuroinflammation and is accompanied by viral disseminating to the brain parenchyma, which may lead to the development of neuronal death, especially in the cerebral cortex, causing neurological changes and microcephaly. These findings reveal that microglia play an important role in the pathogenesis of congenital ZIKV infection [61].

### Microglia mediate neurological damage caused by HIV

Microglia are the main target of HIV-1 infection in the brain [63]. There are viral particles in the infected microglia, suggesting that they may be a potential virus reservoir [64]. The main features of HIV-1 encephalitis are the formation of multinucleated giant cells, microglia nodules, and macrophage infiltration into the central nervous system [65]. The formation of multinucleated giant cells reflects the infection of HIV-1 microglia in vitro. Active infection of microglia is ultimately associated with astrogliosis, myelin pallor, and neuronal loss [65]. Although there are ample evidences confirming that activated microglia have neuropathic effects in HIV-1 encephalitis, there are data suggesting that microglia may have some neuroprotective effects in the early stages of HIV disease [66].

Molecular and histopathology confirm the reduction of autophagy in the brain of patients with HIV-associated dementia. HIV-1-infected microglia release neurotoxins, cytokines, viral proteins, and glutamate; activate NMDA receptors on neurons; cause calcium influx, inhibit neuronal autophagy; enhance apoptotic pathways; and ultimately lead to neuronal death [67]. Enhancing autophagy of neurons in patients with HIV encephalitis may become a target for future treatment. Microarray analyses were performed using brain tissue samples from HIV encephalitis (HIVE) patients, HIV+ patients without encephalitis (HIV/noE), and HIV− controls. It was found that a large number of microglia genes have undergone significant changes during HIV infection, including immune activation and function, kinases, phosphatases, and pro-/anti-apoptotic and neurotrophic factors, indicating that the microglia function is impaired and has a pro-inflammatory tendency [68].

Human cytomegalovirus (HCMV) can cause congenital encephalitis or cause encephalitis in AIDS patients [69]. In patients with advanced AIDS, HCMV-induced central nervous system infection leads to two different neuropathological patterns: microglial nodular encephalitis (MGNE) and ventriculoencephalitis (VE). Microglial nodular encephalitis consists of diffuse microglial cells, aggregated astrocytes, and giant cells. The formation of microglial nodules is considered to be an important cause of HCMV-related dementia in AIDS patients [70].

Recently, a study hypothesized that, in Rasmussen encephalitis (RE), microglial nodules with an upregulation of TLRs provide an environment for the initiation of the later dominating T cell cytotoxicity. However, in the early stages of viral encephalitis, the interaction between microglial nodules and pathogenic T cells remains unclear [71].

### Microglia and pelia

In the brain, Peli1 is mainly expressed in microglia. In a mouse model of lethal West Nile virus (WNV) infection, Peli1 promotes the production of pro-inflammatory cytokines and chemokines in microglia and promotes the entry of T cells and macrophages into the CNS. Peli1 plays a crucial role in WNV entry and replication in human and murine neurons and microglia (see Fig. 2). Peli1 promotes adhesion, replication, and synthesis of mature virions. Autopsy of patients who died of WNV encephalitis found that Peli1 was highly expressed on WNV-infected neurons and adjacent inflammatory cells. Peli1-deficient microglia and neurons cause inflammation and reduce host susceptibility to lethal encephalitis [72]. Microglia are the main producers of central nervous system inflammatory cytokines IL-6 and TNF-α and chemokines CCL2 and CXCL10 after WNV infection [73]. The Peli1-deficient mice have better resistance to WNV, reducing levels of cytokines and chemokines in the body, including IL-6, TNF-α, IL-1β, and IL-10 and CCL2 and CCL5 [72].

Peli1 expressed in microglia can promote the degradation of TNF receptor-associated factor 3 (Traf3) by regulating TLR/MyD88 signaling and can activate microglia during the induction of experimental autoimmune encephalomyelitis (EAE) [74]. It is well known that microglia respond to viral infection by activating p38MAPK [75]. In WNV-infected Peli1^−/−^ neurons, phosphorylation levels of p38MAPK and p65 decreased, indicating that Peli1 plays a positive regulatory role in NF-κB and p38MAPK activation [72]. Peli1 facilitates WNV replication in microglia and neurons, especially in the latter, which are the major cells infected during in vivo challenge. It also positively mediates NF-κB and/or p38MAPK activation in these cells and boosts a robust production of inflammatory cytokines and chemokines, which attracts more infiltration of inflammatory cells from the periphery and ultimately contributes to lethal WNV encephalitis. Thus, Peli1 synergistically promotes virus dissemination and inflammation in the CNS [72].

## Microglia play an uncertain role

### Microglia and virus-induced Aβ

Under physiological conditions, neurons can produce trace amounts of amyloid-β peptide (Aβ) to maintain synaptic plasticity and memory and play an antimicrobial role. The clearance of Aβ is achieved by phagocytosis of microglia. HSV reactivation would trigger increased Aβ production, activation of microglia, and release of pro-inflammatory cytokines (pathological state) that would sustain microglia activation and initiate a vicious circle of inflammatory responses. Recurrence of this series of events during life would result in amplification and irreversible damage to hippocampus [76]. Studies have shown that Aβ oligomers can bind glycoproteins on the surface of herpesvirus and accelerate Aβ deposition showing protective viral entrapment activity in 5XFAD mouse and 3D human neural cell culture infection models against neurotropic HSV1. This indicates that Aβ fibrils and deposits may be important in protecting the brain from common viral infections [77]. Carbohydrate-binding promotes Aβ fibrillization and leads to protofibril generation on microbial surfaces. Bound protofibrils first inhibit host cell adhesion by pathogens. Then, propagating Aβ fibrils mediate agglutination and sequestration of microbes within fibrillar β-amyloid. When the Aβ oligomer-mediated protective antimicrobial pathway is over-activated, progressive Aβ deposition can cause neuroinflammation, neuropathology, and extensive neuronal death, leading to Alzheimer’s disease (AD), a model called antimicrobial protection hypothesis [77]. Recently, a 16-year study involving more than 33,000 patients found that HSV 1 infection increased the risk of dementia by 2.56 times. And long-term use of anti-herpes drugs seems to reduce the risk of dementia in patients with HSV-1 infection. In general, patients with shorter (< 30 days) or longer (≧ 30 days) durations of anti-herpetic medications were associated with a decreased risk of dementia, and the treatment duration of ≧ 30 days was associated with a lower risk of dementia than those of a duration of < 30 days [78].

Microglia have been involved in AD for more than 100 years. Nissl and Alzheimer first described amoeba-like glial cells in various neurological states in 1904 and 1910. This amoeba-like glial cell was later confirmed to be a microglia. Microglia were activated in amyloid plaque-enriched areas in Alzheimer’s disease brains [79]. Some findings reveal that microglia serve as important physiological functions in learning and memory by promoting learning-related synapse formation through brain-derived neurotrophic factor (BDNF) signaling [80]. In the AD mouse model, a disease-associated microglia (DAM) subtype was found. DAM selectively aggregates around Aβ and exerts phagocytosis. Immunohistochemical staining of mice and human brain slices shows DAM with intracellular/phagocytic Aβ particles [81]. The accumulation of microglia around the Aβ plaque is critical to establishing a physical barrier that limits the spread of plaque and protects surrounding neurons from the toxic effects of the plaque [82].

Interestingly, in a mouse model lacking triggering receptor expressed on myeloid cells 2 (TREM2), microglia could not accumulate around Aβ and the survival and proliferation of microglia around the plaque is impaired. At the same time, the migration of microglia to the lesion is impaired [83, 82]. The microglia lacking TREM2 has metabolic disorders, which are characterized by defects in glycolysis, ATP levels, and anabolism [83]. At the same time, the signaling pathway of TREM 2 is essential for phagocytosis of microglia, and the deletion of TREM 2 leads to aggravated pathology [84]. More malnourished neurons were observed in the brain sections in patients carrying AD-associated TREM2 variants [85]. In contrast, it has been reported that when microglia respond to Aβ plaques, it activates inflammatory bodies, leading to the escalation of inflammation and the release of apoptosis-associated speck-like protein containing a CARD (ASC) specks from inflammasomes, which in turn may initiate the seeding of new plaques [86]. Moreover, excessive activation of microglia in the early stages of AD may cause excessive synaptic pruning, leading to cognitive impairment [87]. In summary, HSV-1 infection accelerates the deposition of Aβ in the brain and activates microglia. And the role of Aβ during viral encephalitis remains obscure and requires additional study.

### Microglia and RIPK3

Necroptosis is a programmed cell death coordinated by receptor-interacting protein kinases 1 (RIPK1) and 3 (RIPK3). Mixed lineage kinase domain-like protein (MLKL) is an executioner protein that participates in the formation of an apoptotic complex that promotes cell death through cell membrane disruption and cell rupture [88]. Studies have shown that the RIPK3 signaling pathway can control a variety of viral infections, including HSV-1. Murine but not human RIP1/RIP3 directly senses HSV-1 ICP6 to initiate necroptosis [89]. In the WNV virus infection model, it was found that RIPK 3 inhibits viral pathogenesis by cell death-independent neuroinflammation, mice lacking RIPK 3, or RIPK 1 kinase activity, but not those lacking mlkl or mlkl, and caspase-8 increased susceptibility to lethal WNV infection. During WNV infection of neurons, RIPK3 is activated in neurons which promotes the expression of chemokines CCL2, CXCL10, etc. and recruits infiltration of immune cells including T cells, mononuclear macrophages, and antigen presenting cells which exert antiviral effects. This process does not affect the number of microglia in CNS [90]. What role does the RIPK3 signaling pathway in microglia play in viral encephalitis? Further exploration is needed.

## Future perspectives

Most of the experiments on microglia and viral encephalitis remain in the experimental stage, and many mechanisms need to be further explored before being applied to the clinic. Although microglia can recognize ATP released by infected neurons through P2Y12 and are recruited around neurons, whether there are more other potent chemokines, further exploration is needed. Although IFN-1 plays an antiviral role in most encephalitis, the optimal IFN-1 concentration and optimal application time are still undetermined. Microglia can induce autophagy to exert antiviral effects, but there is insufficient evidence. Microglia produce inflammatory and chemokines and induce adaptive immune responses. Microglia resident in different brain regions have specific gene expression profiles, and different brain regions have different susceptibility to viruses. The relationship between the two remains undetermined, and it is expected that the innate immune response to the virus will be enhanced by changing the gene expression profile of microglia in the future. In WNV infection, microglia mediate presynaptic terminal loss in the hippocampus though recognizing C3 by C3R, C3, or C3R-specific antibodies may block this process. Microglia inhibit autophagy in patients with HIVE, aggravating CNS injury. How to inhibit or delete the function of microglia or how to enhance the autophagy of neurons will become the direction of future research. There is a strong relationship between fetal cerebellar malformation and activation of microglia in ZIKV infection, and it is necessary to develop microglia inhibitors that are safe for pregnant women and fetuses. Pelia of microglia promotes viral replication in neurons, and moderate blockade of pelia may become a target for future treatment. In viral encephalitis, Aβ produced by neurons increases the risk of dementia in patients. Studies show that prolonging antiviral time will reduce the risk of dementia. The interaction between Aβ and activated microglia in patients with viral encephalitis is unknown. It is unknown whether moderately enhancing the phagocytosis of microglia can reduce the risk of dementia. It needs further research. Traditionally, activation of RIPK3 will result in the formation of an apoptotic complex that mediates cell death. Recent studies have shown that in viral encephalitis, activation of neuronal RIPK3 exerts an antiviral effect in a death-independent pathway, and the RIPK3 pathway of microglia requires further investigation.

## Conclusions

Microglia account for 10% of the total number of cells in the adult central nervous system [91]. In a healthy CNS, microglia are not “resting” but in a highly active “surveillance and rapid response” state [92]. Microglia processes are dynamic, continuously scanning the environment and sampling it [93]. They are able to detect changes in pH, purines, cytokines, chemokines, amino acids, and inorganic compounds [94]. Microglia are derived from the primitive macrophages of the yolk sac, which migrate to the developing central nervous system. They have the ability to self-renew, thus maintaining their number throughout life without any input from the bone marrow-derived precursor cells [95, 96]. Microglia can respond to neurons, which in turn affects neuronal activity. Microglia are involved in the programmed death of neurons and the apoptosis and clearance of new neurons during development. The activity of microglia promotes the pruning, elimination, and maturation of synapses [92]. Microglia serve important physiological functions in learning and memory by promoting learning-related synapse formation through BDNF signaling [80]. The phagocytic activity of microglia is essential for the clearance of senescent cells and debris [97], slowing the toxic effects of amyloid-β [98]. Microglia are not only important cells that maintain the homeostasis of CNS, but also respond to injury, infection, and neurodegeneration through proliferation and altered transcription and morphology [99, 92]. Microglia are often touted as the first responder to CNS infection and respond quickly to injury [93]. However, depending on the stimulus received, this activation profile is also different and may result in harmful or beneficial effects [100, 6]. HSV-1 can be treated, but can WNV or enteroviruses? The high morbidity and high morbidity rate of viral encephalitis have caused widespread concern. Although traditional antiviral therapy is effective, there are still many shortcomings. Research on microglia and viral encephalitis provides new targets for treatment. Microglia play a unique role in different encephalitis. The current research on microglia and viral encephalitis remains at the experimental stage. In the future, depending on the type of encephalitis, rationally enhancing or inhibiting the activation of microglia will greatly improve the therapeutic effect of encephalitis, improve the prognosis of traditional antiviral therapy, and reduce the sequelae of encephalitis.

## Abbreviations

AD: Alzheimer’s disease
AIDS: Human acquired immunodeficiency syndrome
AMPs: Antimicrobial peptides
ASC: Apoptosis-associated speck-like protein containing a CARD
Aβ: Amyloid-β peptide
BBB: Blood-brain barrier
BDNF: Brain-derived neurotrophic factor
BMECs: Brain microvascular endothelial cells
cGAMP: Cyclic-GMP–AMP
cGAS: GMP–AMP synthase
CMV: Cytomegalovirus
CNS: Central nervous system
CSF: Cerebrospinal fluid
CSF1R: Colony-stimulating factor 1 receptor
DAM: Disease-associated microglia
dSTING: Drosophila stimulator of interferon genes
EAE: Experimental autoimmune encephalomyelitis
EEG: Electroencephalographic
elav-gal4: A neuron-specific gal4 driver
HCMV: Human cytomegalovirus
HIV/noE: HIV+ patients without encephalitis
HIVE: HIV encephalitis
HSE: Herpes simplex encephalitis
HS-gal4: Heat shock-inducible gal4
HSV: Herpes simplex virus
IFN-1: Type 1 interferon
IFN-α: Interferon α
IFNαR: IFN-α receptor
IFNαR: Interferon receptor
IFN-β: Interferon β
IL-6: Interleukin 6
IL-1β: Interleukin 1β
IL-34: Interleukin 34
IP-10: IFN-γ inducible protein 10 kD
IRF3: IFN regulatory factors 3
IRF7: IFN regulatory factors 7
ISGs: IFN-stimulated genes
MAVS: Mitochondrial antiviral signaling
MCP-1: Monocyte chemoattractant protein 1
MDA-5: Melanoma differentiation-associated protein 5
MGNE: Microglial nodular encephalitis
MHV: Mouse hepatitis virus
MLKL: Mixed lineage kinase domain-like protein
NMDA: *N*-methyl-D-aspartic acid receptor
P2Y12^−/−^: P2Y12 deficiency
PAMPs: Pathogen-associated molecular patterns
Peli1: E3 ubiquitin ligase Pellino 1
PRR: Pattern recognition receptors
RE: Rasmussen encephalitis
Repo-gal4: A glia-specific gal4 driver
RIG-I: Retinoic acid-inducible gene-1
RIPK1: Receptor-interacting protein kinases 1
RIPK3: Receptor-interacting protein kinases 3
RLRs: RNA-sensing RIG-like receptors
STAT 1/2: Signal transducer and activator of transcription 1/2
STING: Stimulator of type I IFN genes
TLR3: Toll-like receptor 3
TNFR1: TNF receptor type 1
TNF-α: Tumor necrosis factor α
Traf3: TNF receptor-associated factor 3
TREM2: Triggering receptor expressed on myeloid cells 2
TRIF: TIR domain-containing adaptor inducing IFN-β
VCAM-1: Vascular cell adhesion molecule 1
VE: Ventriculoencephalitis
VLA-4: Leukocyte adhesion molecule
VSV: Vesicular stomatitis virus
VZV: Varicella–zoster virus
WNV: West Nile virus
ZIKV: Zika virus
